# Urine Trefoil Factors as Prognostic Biomarkers in Chronic Kidney Disease

**DOI:** 10.1155/2018/3024698

**Published:** 2018-04-03

**Authors:** Toshio Yamanari, Hitoshi Sugiyama, Keiko Tanaka, Hiroshi Morinaga, Masashi Kitagawa, Akifumi Onishi, Ayu Ogawa-Akiyama, Yuzuki Kano, Koki Mise, Yasukazu Ohmoto, Kenichi Shikata, Jun Wada

**Affiliations:** ^1^Department of Nephrology, Rheumatology, Endocrinology and Metabolism, Okayama University Graduate School of Medicine, Dentistry and Pharmaceutical Sciences, Okayama, Japan; ^2^Department of Human Resource Development of Dialysis Therapy for Kidney Disease, Okayama University Graduate School of Medicine, Dentistry and Pharmaceutical Sciences, Okayama, Japan; ^3^Institute of Biomedical Innovation, Otsuka Pharmaceutical Co., Ltd., Tokushima, Japan; ^4^Center for Innovative Clinical Medicine, Okayama University Hospital, Okayama, Japan

## Abstract

**Introduction:**

Trefoil factor family (TFF) peptides are increased in serum and urine in patients with chronic kidney disease (CKD). However, whether the levels of TFF predict the progression of CKD remains to be elucidated.

**Methods:**

We determined the TFF levels using peptide-specific ELISA in spot urine samples and performed a prospective cohort study. The association between the levels of urine TFFs and other urine biomarkers as well as the renal prognosis was analyzed in 216 CKD patients (mean age: 53.7 years, 47.7% female, 56.9% with chronic glomerulonephritis, and mean eGFR: 58.5 ml/min/1.73 m^2^).

**Results:**

The urine TFF1 and TFF3 levels significantly increased with the progression of CKD stages, but not the urine TFF2 levels. The TFF1 and TFF3 peptide levels predicted the progression of CKD ≥ stage 3b by ROC analysis (AUC 0.750 and 0.879, resp.); however, TFF3 alone predicted CKD progression in a multivariate logistic regression analysis (odds ratio 3.854, 95% confidence interval 1.316–11.55). The Kaplan-Meier survival curves demonstrated that patients with a higher TFF1 and TFF3 alone, or in combination with macroalbuminuria, had a significantly worse renal prognosis.

**Conclusion:**

The data suggested that urine TFF peptides are associated with renal progression and the outcomes in patients with CKD.

## 1. Introduction

Chronic kidney disease (CKD) is defined as having either glomerular filtration rate (GFR) < 60 mL/min/1.73 m^2^ or markers of kidney damage for at least 3 months or both [1, 2]. CKD with multifactorial etiology leading to end-stage renal disease (ESRD) is a significant concern, given the increasing numbers of such patients worldwide [3]. CKD is not only associated with an elevated risk of ESRD but also with cardiovascular disease and mortality, even with a slight decline in the GFR [4, 5]. A lower estimated GFR (eGFR) and severe albuminuria independently predict ESRD and mortality in patients with CKD [6]. Several reports have identified and validated novel biomarkers in CKD patients in order to better identify those at high risk of a rapid loss of the renal function [7].

The mammalian trefoil factor family (TFF) peptides consist of a three-looped structure of cysteine residues, known as the trefoil domain, and the family comprises three members in mammals: TFF1, TFF2, and TFF3 [8, 9]. TFF1 and TFF3 contain one trefoil domain, while TFF2 contains two. TFF1 and TFF3 can dimerize to homodimers through a seventh cysteine residue [10]. These small peptides, with a molecular weight of approximately 7 kDa, are secreted by mucus-producing cells in the gastrointestinal tract and are involved in mucosal surface maintenance and repair [11, 12]. They are also secreted by epithelial cells of multiple tissues, including tubular epithelial cells of kidney [13], through a seventh cysteine residue located near the C-terminus.

In the human urinary tract, TFF3 is detected as the most abundant form followed by TFF1 [14]. In rodent models, urine TFF3 markedly reduced after acute renal toxicity [15], and it has already been proposed as a urine biomarker for kidney toxicity in preclinical stages [16]. Higher urine levels of TFF3 were shown to be associated with incident CKD in community-based populations [17]; however, they were not associated with incident CKD or albuminuria in another prospective cohort of Framingham Heart Study participants [18]. In recent studies in patients with CKD, increased levels of urine TFF1 [19] and urine TFF2 [20] have been reported in early CKD stages, whereas urine TFF3 levels are increased in later CKD stages [19, 21].

Given the above conflicting findings, whether or not urine TFF levels can be used to predict the renal outcome is still uncertain in patients with CKD. We therefore examined the urine levels of TFF and investigated the relationship between urine TFF and the renal progression and outcomes in patients with CKD.

## 2. Material and Methods

### 2.1. Subjects

The subjects in this study were outpatients who had visited the Renal Unit of Okayama University Hospital between February 2009 and January 2011. All patients were diagnosed with CKD according to their eGFR and the presence of kidney injury, as defined by the National Kidney Foundation K/DOQI Guideline [22]. Hypertension was defined as systolic blood pressure (SBP) ≥ 140 mmHg or diastolic blood pressure (DBP) ≥ 90 mmHg or the use of antihypertensive drugs. The eGFR was calculated according to the simplified version of the Modification of Diet in Renal Disease formula [eGFR = 194 × (sCr)^−1.094^  × (age)^−0.287^ (if female × 0.739)] [23]. All procedures in the present study were carried out in accordance with institutional and national ethical guidelines for human studies and guidelines proposed in the Declaration of Helsinki. The ethics committee of Okayama University Graduate School of Medicine, Dentistry and Pharmaceutical Sciences approved the study (number 522 and revision number 2063). Written informed consent was obtained from each subject. This study was registered with the Clinical Trial Registry of the University Hospital Medical Information Network (registration number UMIN000010140).

According to the protocol, we excluded any patients with established atherosclerotic complications (coronary artery disease, congestive heart failure, or peripheral vascular disease). Patients with nephrotic syndrome, acute kidney injury, acute infection, and malignancy including gastric cancer [24, 25], active gastrointestinal diseases including gastroenteritis and peptic ulcers, or liver cirrhosis [26] at entry were excluded (Supplementary Figure S1).

### 2.2. Study Samples

All urine samples were obtained from patients from spot urine in the morning [27]. Samples were spun at 2,000*g* for 5 minutes in a refrigerated centrifuge, and the supernatants were immediately transferred to new screw-top cryovial tubes and frozen at −80°C. All urine aliquots used in this biomarker study had undergone no previous freeze-thaw cycle. Samples for this study were obtained from 216 participants who were free of ESRD at the time of urine collection.

### 2.3. Biomarker Measurements and Other Clinical Parameters

The mean storage duration between collection and measurement was a median of 29 months (interquartile range, 28–31 months). The TFF peptide (TFF1, TFF2, and TFF3) concentrations were measured using an ELISA system, as described previously [24, 25]. Antisera were prepared from rabbits immunized with human TFFs. Purified polyclonal antibodies (TFF1: OP-22203, TFF2: OP-20602, and TFF3: OPP-22303) were coated onto a 96-well microtiter plate, and the plates were blocked with 0.1% bovine serum albumin/phosphate-buffered saline (PBS). After the blocking solution was removed, 100 *μ*L of assay buffer (1 mol/L NaCl/0.1% bovine serum albumin/PBS) and 50 *μ*L of sample or human TFF standard were added to the wells. After incubation overnight at room temperature, the plate was washed, and diluted biotin-labeled anti-TFF polyclonal antibodies (TFF1: biotin-OPP22205, TFF2: biotin-OPP20601, and TFF3: biotin-OPP22305) were added to each well. After incubation for 2 h, the plate was washed, and diluted horseradish peroxidase-conjugated streptavidin (Vector Laboratories, Burlingame, CA, USA) was added to each well, followed by a further 2 h incubation at room temperature, after which the plate was washed. Tetramethylbenzidine (TMB) solution (Scytek Laboratories, Inc., West Logan, UT, USA) was then added, stop solution (Scytek Laboratories, Inc.) was added 10 min later, and the absorbance at 450 nm was measured. Concentrations of human TFFs in the samples were calculated from a standard curve constructed from recombinant human TFFs. The assay sensitivities for TFF1, TFF2, and TFF3 were 7, 30, and 30 pg/mL, respectively. Each TFF antibody reacted specifically and showed no cross-reactivity for the other TFFs [24, 25]. The performance characteristics of the ELISA are shown in Supplementary Table S1 and Supplementary Figure S2.

The concentrations of clinical parameters were measured using routine laboratory methods (SRL, Inc., Okayama, Japan). The urinary levels of albumin, *α*1-microglobulin (*α*1-MG), *β*2-microglobulin (*β*2-MG), and N-acetyl-*β*-D- glucosaminidase (NAG) were also determined (SRL, Inc.). The serum and urinary creatinine levels were measured according to the enzymatic colorimetric method. Each subject's arterial blood pressure was measured by a physician after a 10-minute resting period to obtain the systolic and diastolic blood pressures (SBP and DBP, resp.). The mean blood pressure (MBP) was calculated as DBP + (SBP − DBP)/3 [28].

### 2.4. Outcomes and Follow-Up

The primary outcome was CKD progression, defined as a composite endpoint of incident ESRD (recipient of maintenance dialysis or kidney transplant) or doubling of serum creatinine [29]. Patients were prospectively followed up for a median period of 1097 days (interquartile range, 794–1244 days). Patients were followed by review of the medical record or telephone interview at least twice a year until March 31, 2013. Death and loss to follow-up were considered censoring events.

### 2.5. Statistical Analyses

Statistical analyses were performed using the JMP software package (release 11; SAS Institute, Cary, NC, USA). Data are expressed as the mean ± standard deviation for continuous parametric data, median and interquartile range for continuous nonparametric data, and frequencies for categorical data. A linear regression analysis of the data at baseline was performed using the least-squares method. Variables showing a positively skewed distribution were transformed using the natural logarithm (ln). Differences between groups were analyzed using Student's* t*-test and the Mann–Whitney *U* test as appropriate. Receiver operating characteristic (ROC) curves were constructed to determine the optimum sensitivity and specificity, and the area under the curve (AUC) was calculated [30]. A multivariable logistic regression analysis was performed to determine the predictors [28]. The *p* values, odds ratios, and corresponding two-sided 95% confidence intervals for the predictors are presented [31]. A Kaplan-Meier analysis and the log-rank statistic were used to explore the effect of urine biomarker levels on the renal endpoint-free survival [29, 31]. Renal survival times were censored only when patients died, underwent maintenance dialysis or kidney transplantation, were lost to follow-up monitoring, or completed the study. The renal survival was calculated from the date of urine sample collection. A value of *p* < 0.05 was considered to be statistically significant.

## 3. Results

### 3.1. Urine TFF Levels in Early, Middle, and Later CKD Stages

**Figure 1:**
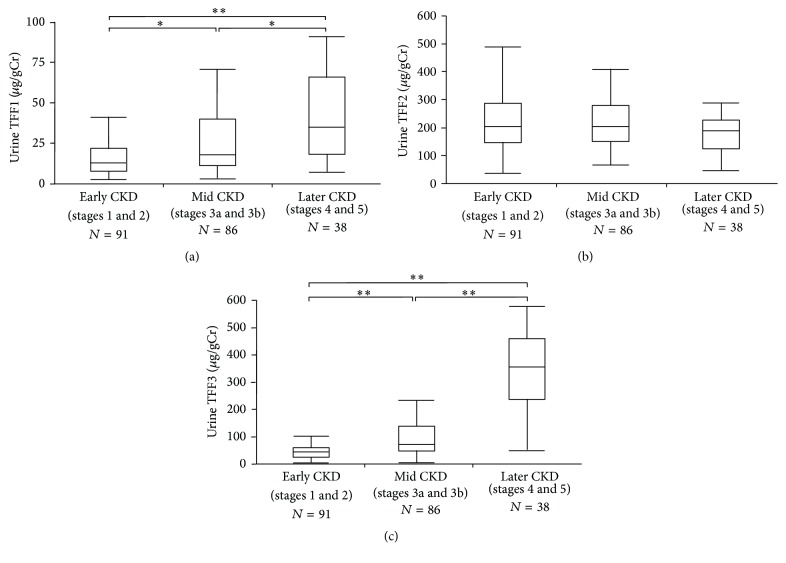
Box and line plots showing the levels of urine TFF according to the CKD stages. The levels of both urine TFF1 (*μ*g/gCr) (a) and TFF3 (*μ*g/gCr) (c) increased along with advancement of CKD stages, while those of urine TFF2 (*μ*g/gCr) did not (b). *∗* and *∗∗* indicate *p* < 0.005 and *p* < 0.0001, respectively. The boxes denote the medians and 25th and 75th percentiles. The lines mark the 5th and 95th percentiles.

A total of 216 CKD patients with a mean age of 53.7 years were included in the study (Table 1). More than half of background causes of CKD included glomerulonephritis (56.9%). The median urine TFF1, TFF2, and TFF3 levels were 16.6, 199.7, and 65.3 *μ*g/gCr, respectively. The baseline characteristics are shown according to early (stages 1 and 2), middle (stages 3a and 3b), and later (stages 4 and 5) CKD stages (Table 1). Of note, the concentrations of both urine TFF1 and TFF3 significantly increased with progression of CKD stages; however, those of urine TFF2 did not (Figure 1). Concentrations of other urine markers of tubular injury, including *α*1-MG, *β*2-MG, and NAG, also increased with CKD progression (Table 1). Regarding the relationships among urine TFF peptides and other tubular injury markers, TFF3 correlated well with *α*1-MG, *β*2-MG, and NAG, and TFF1 correlated well with *α*1-MG and *β*2-MG, but TFF2 did not exhibit significant correlations with any of these markers (Table 2). The associations among mutual TFF peptides in urine were significant except for those between TFF2 and TFF3. The correlations between urine TFF peptides and age were significant (Supplementary Table S2). The data of urine TFF levels in healthy subjects is also shown in Supplementary Table S3.

### 3.2. Predictors of CKD Progression ≥ 3b (eGFR < 45 mL/min/1.73 m^2^) in Urine Biomarkers

The parameter with the largest area under the ROC curve (AUC) for predicting the progression of CKD ≥ 3b was urine TFF3 (0.879), followed by urine *α*1-MG (0.874) (Table 3). Regarding the other urine biomarkers, the AUCs of *β*2-MG, TFF1, albumin, and NAG were also significant. Serum factors such as hemoglobin and uric acid were significant as well for predicting the CKD progression, as expected in a typical CKD cohort (Supplementary Table S4). In an analysis with the ratio of urine TFF3 to other parameters, the AUC of the ratio of urine TFF3 to urine TFF2 was the largest (0.859) (Supplementary Table S5). In a multivariate logistic regression analysis, higher levels of urine TFF3 (more than the median value, 65.3 *μ*g/gCr) and urine *α*1-MG (more than the median value, 3.81 mg/gCr) at the start of the study were significantly associated with the CKD progression (Table 4).

### 3.3. Prediction of the Renal Survival by Urine TFF

**Figure 2:**
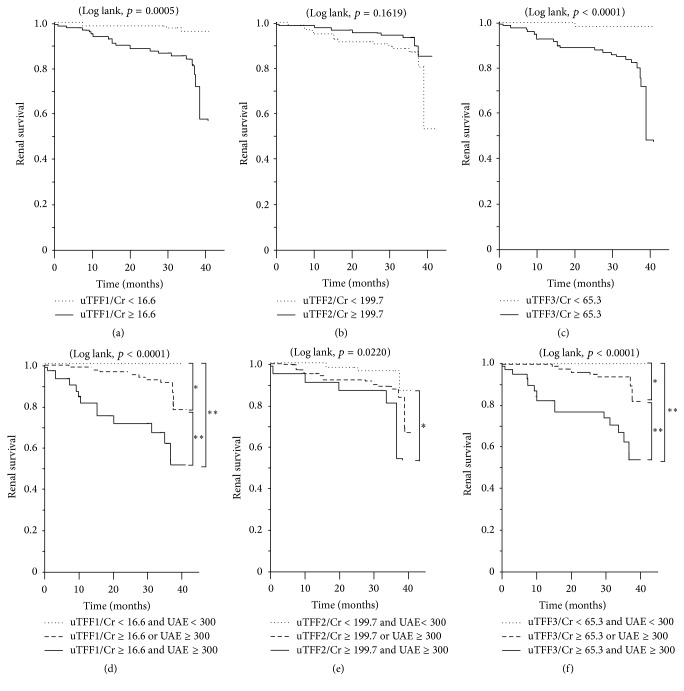
The renal survival categorized by TFF alone (a–c) or by their combination with albuminuria (d–f). The median value of urine TFF1 (*μ*g/gCr) (a) or TFF3 (*μ*g/gCr) (c) predicted the three-year renal endpoint-free survival, while urine TFF2 (*μ*g/gCr) did not (b). The combination of urine TFF1 (d) or TFF3 (f) with albuminuria clearly separated the three-year renal endpoint-free survival of CKD patients, while that of urine TFF2 with albuminuria had a less obvious effect (e). (d) uTFF1/Cr < 16.6 and UAE < 300, *n* = 89 (41.2%), uTFF1/Cr ≥ 16.6 or UAE ≥ 300, *n* = 93 (43.1%), uTFF1/Cr ≥ 16.6 and UAE ≥ 300, *n* = 34 (15.7%). (e) uTFF2/Cr < 199.7 and UAE < 300, *n* = 78 (36.1%), uTFF2/Cr ≥ 199.7 or UAE ≥ 300, *n* = 113 (52.3%), uTFF2/Cr ≥ 199.7 and UAE ≥ 300, *n* = 25 (11.6%). (f) uTFF3/Cr < 65.3 and UAE < 300, *n* = 95 (44.0%), uTFF3/Cr ≥ 65.3 or UAE ≥ 300, *n* = 81 (37.5%), uTFF3/Cr ≥ 65.3 and UAE ≥ 300, *n* = 40 (18.5%). *∗* and *∗∗* indicate *p* < 0.01 and *p* < 0.0001, respectively. UAE, urinary albumin excretion (mg/gCr).

**Figure 3:**
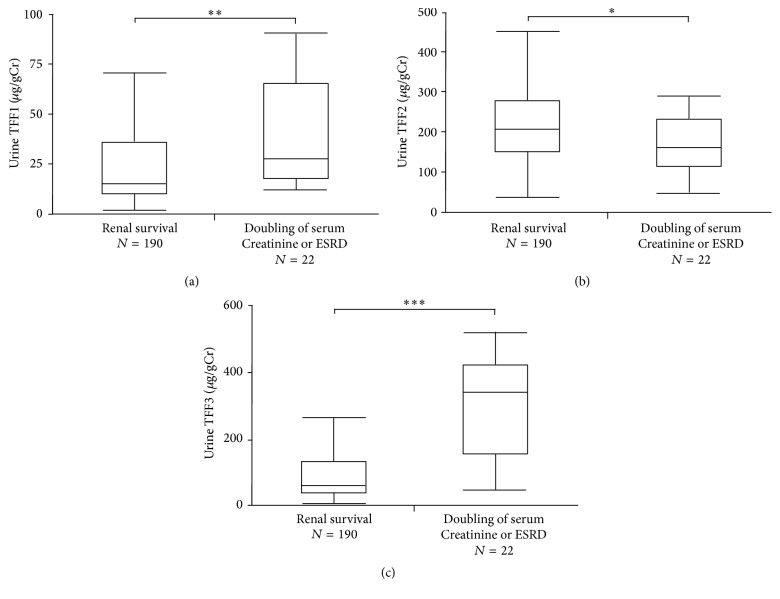
The levels of urine TFF for the renal endpoint group and the renal survival group. The renal endpoint group (right box and line plot) had higher levels of urine TFF1 (a) and TFF3 (c) but lower levels of urine TFF2 than the renal survival group (b). *∗*, *∗∗*, and *∗∗∗* indicate *p* < 0.05, *p* < 0.001, and *p* < 0.0001, respectively. The boxes denote the medians and 25th and 75th percentiles. The lines mark the 5th and 95th percentiles.

To investigate whether or not the baseline urine TFF levels predict the subsequent renal survival in CKD patients, we categorized the patients into groups by the level of each TFF (median value, *μ*g/gCr) or in their combination with albuminuria (<300 or ≥300 mg/gCr) in Kaplan-Meier survival analyses (Figure 2). We observed a significant difference in the three-year renal endpoint-free survival when patients were divided into groups according to the median value of urine TFF1 or TFF3 (Figure 2(a)or 2(c)). In contrast, we observed no significant difference in the three-year renal survival when patients were divided into groups by the median value of urine TFF2 (Figure 2(b)). Combining urine TFFs, which are suspected tubular injury markers, with albuminuria, which mainly reflects glomerular injury, the three-year renal endpoint-free survival probabilities were 100.0% (d), 87.3% (e), and 100.0% (f) for lower TFF1, TFF2, and TFF3 levels (less than the median value) and lower albuminuria (<300 mg/gCr); 78.7% (d), 67.2% (e), and 81.7% (f) for higher albuminuria (≥300 mg/gCr) alone or higher TFF1, TFF2, or TFF3 alone; and 51.8% (d), 54.0% (e), and 53.4% (f) for both higher albuminuria and higher TFF1, TFF2, or TFF3 levels (Figures 2(d)–2(f)). During the 36 months of follow-up, 22 patients exhibited doubling of serum creatinine (*n* = 14) or ESRD requiring renal replacement therapy (*n* = 8). Compared with the remaining 190 patients (the renal survival group), the renal endpoint group had significantly higher levels of urine TFF1 and TFF3 but significantly lower levels of urine TFF2 (Figure 3).

The analyses without creatinine correction of the levels of urine TFF according to the CKD stages, for the renal survival and for the renal endpoint group or the renal survival group, are shown in Supplementary Figures S3, S4, and S5, respectively.

## 4. Discussion

In this study, we measured the urine TFF levels in early, middle, and later CKD patients and determined the relationships between the urine TFF level and the CKD progression and outcomes. Based on analyses of urine samples from CKD patients, we found that (1) the TFF1 and TFF3 levels significantly increased with progression of CKD stages, while TFF2 did not; (2) TFF3 to a better degree and TFF1 to a lesser degree correlated with the decline in the eGFR and other urine markers of tubular injury, including *α*1-MG and *β*2-MG, as well as other family peptides TFF1 and TFF3, respectively; (3) TFF1 and TFF3 were significant predictors of the progression of CKD ≥ 3b in an ROC analysis, and TFF3 alone, but not TFF1 or TFF2, was a significant predictor in a multiple logistic regression analysis; and (4) in a survival analysis, TFF1 and TFF3 either alone or in combination with the level of albuminuria were a significant predictor of the renal outcome in patients with CKD.

We showed that the urine levels of both TFF1 and TFF3 significantly increased with the progression of CKD, while the urine levels of TFF2 did not (Figure 1). Regarding urine TFF3, Du et al. reported findings consistent with our own on urine TFF3 in CKD patients [21]. However, as for urine TFF1, Lebherz-Eichinger et al. reported that urine TFF1 levels increased in the early stages of CKD and declined with disease progression without significant changes in the fractional excretion of TFF1 [19], which is inconsistent with our data on urine TFF1. TFF2 was the first TFF to be identified and characterized [8, 9]. In the human urinary tract, TFF3 is detected as the most abundant form followed by TFF1 [14], while urine TFF2 and TFF3 are increased in patients with nephrolithiasis [14]. A recent study evaluated the urine TFF2 levels in patients with CKD [20]. Urine TFF2 concentrations were significantly higher in early or middle CKD stages than in later CKD stages and predicted early CKD stages in an ROC analysis, but without significant changes in the fractional excretion of TFF2 among CKD stages [20], which is also inconsistent with our data on urine TFF2. Further studies will be required to clarify these inconsistencies in data on urine TFF1 and TFF2 levels at different CKD stages.

The origin of urine TFF peptides has yet to be fully elucidated. TFF3 mRNA is expressed in the cortex of the human kidney [14], in contrast to genes that encode other TFF members. Elevated levels of TFF3 were also found in urine from patients with incident chronic kidney disease as part of a nested case-control study [17], as well as in serum of patients with CKD stages 1–5 [19, 21]. Cultured human proximal tubular epithelial cell line HK-2 can synthesize and excrete TFF3 after exposure to immunoglobulin *λ* light chain, but not after exposure to fatty acid-free human serum albumin [32]. The promotor region of human TFF3 has the STAT3 binding site critical for the self-induction of TFF3 [33] as well as the NF-*κ*B binding site [34]. Possible triggers for the increase in TFF3, at least in part, may include inflammation via the transcription factors STAT3 and NF-*κ*B, both of which are proposed as central regulators of CKD progression [35, 36].

The exact role of TFF in the kidney is still uncertain. TFF3, also known as intestinal trefoil factor (ITF), a peptide expressed in goblet cells of the intestines, colon, and kidney [37], plays essential functions in both mucosal surface maintenance and repair [12]. By inhibiting apoptosis and promoting the survival and migration of epithelial cells into lesions, TFF3 facilitates the restoration of intestinal epithelium as a protective barrier against injury [38, 39]. TFF3 also plays a role in inducing airway epithelial ciliated cell differentiation [40]. Systemic TFF3 KO mice developed normally and were grossly indistinguishable from their wild-type littermates without apparent renal abnormalities but exhibited poor epithelial regeneration of mucosa after intestinal injury [41]. TFF3 might play a role in the repair of tubular epithelium in kidney, similar to its role in the gastrointestinal tract. Examining conditional knockout mice of TFF3 specific to renal tubular epithelial cells may help clarify the precise function of TFF3 in the kidney.

The kidney tubules of the outer stripe of the outer medulla are a major site of* tff3* mRNA expression in rodents [15]. Histochemical localization using a labeled TFF3 fusion protein detected binding sites in the collecting ducts of the kidney [42], and aging was correlated with a decreased renal expression of* tff3* transcript in rodents [43]. In the normal human kidney, TFF3 has been found in proximal and single distal tubular cells as well as in collecting duct cells from which a small amount of TFF1 is also secreted by immunohistochemistry, while only TFF3 is detectable by a Western blot analysis in the medulla [14]. In the collecting ducts of the medulla, TFF1 and TFF3 are constituents of the mucus layer [14]. These reports suggest that the increases in the TFF3 and TFF1 in urine reflect their excretion from the urinary tract of CKD patients, not merely their leakage from serum.

The renal distribution of TFF3 protein in CKD patients is very scant. Immunohistochemistry of renal biopsy specimens showed aberrant expression of TFF3, which was localized to the tubular epithelial cells in the renal cortex but not to the glomeruli, arterioles, or interstitium [21]. The recent genome-wide association study in the Framingham Heart Study revealed an association between TFF3 and LRP2 with multiple variants independently associated with urinary TFF3 levels [44]. Since* lrp2* encodes megalin, a multiligand endocytic receptor localized in the renal proximal tubule, TFF3 might be a megalin ligand, such like *α*1-MG or *β*2-MG [45], leading to altered tubular handling of TFF3 in the presence of the variants. In acute kidney injury (AKI) of animal models, a decrease in both urine TFF3 levels and renal TFF3 staining was observed in nephrotoxin-treated rodents [15], suggesting a gene regulatory response of TFF3 to tubular toxicity in this setting. In AKI of patients with acute decompensation of liver cirrhosis, urine TFF3 levels are significantly increased, particularly in acute tubular necrosis, compared to patients without AKI [26].

In the survival analysis of this study, TFF1 and TFF3 either alone or in combination with the level of albuminuria were found to be a significant predictor for the disease progression and renal outcome in patients with CKD (Figure 2). In an analysis of a panel of 14 urine biomarkers for incident kidney disease and the clinical outcome in the Framingham Heart Study participants, urine TFF3 levels predicted the all-cause mortality and death with coexistent kidney disease but not with incident CKD or albuminuria, although it did not investigate the renal outcome of doubling of the serum creatinine level or incident ESRD [18].

The ROC analysis of this study showed that urine TFF3 was a useful biomarker for predicting the progression of CKD ≥ 3b. Although other biomarkers, such as urine *α*1-MG, urine *β*2-MG, and hemoglobin, were also shown to be good predictors (Table 3 and Table S4), the AUC of urine TFF3 was the largest among these biomarkers, and the invasiveness of its measurement is lower than those of other serum biomarkers. These findings underscore the usefulness of measuring the urine TFF3 levels.

Our study has several limitations and strengths that should be kept in mind when interpreting the results. First, we lacked enough data in patients with diabetic nephropathy, which was the most frequent cause of ESRD in modern countries. However, including diabetic patients in the CKD cohort might have influenced the TFF levels, as other biomarkers such as serum Klotho are lower in diabetic patients than in nondiabetic patients [46]. Second, several methods for measuring the TFF levels have been established using in-house ELISA assays, such as in this study and others [21], or are commercially available in ELISA kits [19] or bead immunoassay platforms [17, 18]. Previously published data have reported TFF3 concentrations in the urine of normal and diseased individuals to span the range between 0.03 and 7.0 *µ*g/mL [47, 48]. The validation of the TFF assay will be of great importance in the near future such as a paper-based assay which can be quickly and inexpensively performed [48]. Third, relatively few patients reached the outcome, which might have influenced the results of this study to some extent. Fourth, the precise expression of TFF in the kidney tissue of patients with CKD was not investigated in this study, although a previous report showed localization of TFF3 in the renal tubular epithelial cells, but not in the glomeruli, arterioles, or interstitium in renal biopsy specimens of 23 patients with CKD [21].

## 5. Conclusions

Our data showed that urine TFF peptides are associated with other urine tubular injury markers and the renal outcomes in patients with CKD. Further studies are required to elucidate the precise localization and function of TFF in the human kidney and its role in the progression in CKD patients. Interventions that can modulate the level of urine TFF in such patients may be useful, since improving the outcome is the ultimate goal of biomarker studies.

## Figures and Tables

**Table 1:** Baseline characteristics of the study subjects.

	All patients	Early CKD (stages 1 and 2)	Mid CKD (stages 3a and 3b)	Later CKD (stages 4 and 5)	*p*
*N*	216	91 (42.3%)	86 (40.0%)	38 (17.7%)	
Age (year)	53.7 ± 18.1	41.0 (28.0–58.0)	64.0 (47.8–72.0)	64.5 (54.8–75.3)	<0.0001
Male gender, *n* (%)	113 (52.3%)	41 (45.1%)	48 (56.5%)	23 (60.5%)	0.1576
Cause of CKD, *n*					<0.0001
Chronic glomerulonephritis^*∗*^	123 (56.9%)	68 (74.7%)	46 (54.1%)	7 (18.4%)	
Nephrosclerosis	22 (10.1%)	2 (2.2%)	11 (12.9%)	9 (23.7%)	
Diabetic nephropathy	6 (2.8%)	1 (1.1%)	2 (2.4%)	3 (7.9%)	
Others^*∗∗*^	65 (30.1%)	20 (22.0%)	26 (30.6%)	19 (50.0%)	
UAE (mg/gCr)	47.5 (7.8–299.0)	20.8 (5.3–89.0)	71.4 (9.7–359.0)	435.5 (32.9–1375)	<0.0001
eGFR (mL/min/1.73 m^2^)	58.5 ± 30.6	82.1 (70.5–95.9)	46.4 (37.9–54.6)	18.3 (14.9–24.8)	<0.0001
MBP (mmHg)	91.0 ± 10.9	90.6 ± 11.8	91.3 (83.5–96.7)	90 (83.3–99.3)	0.9331
Serum albumin (g/dL)	4.0 ± 0.4	4.3 (4.0–4.5)	3.9 (3.6–4.2)	3.8 (3.4–4.0)	<0.0001
Hemoglobin (g/dL)	12.9 ± 1.74	13.4 (12.5–14.8)	12.9 (12.0–14.2)	10.8 (10.2–11.7)	<0.0001
LDL-cholesterol (mg/dL)	113 ± 34	103 (90–131)	119 (99–143)	99 (74–136)	0.0714
Triglycerides (mg/dL)	133 (96–184)	112 (77–154)	151 (111–232)	141 (97–174)	0.0008
Uric acid (mg/dL)	6.4 ± 1.8	5.7 (4.4–6.3)	6.7 ± 1.5	8.0 (6.7–8.8)	<0.0001
Hemoglobin A1C (%)	5.6 (5.2–5.8)	5.4 (5.1–5.7)	5.6 (5.3–5.9)	5.7 (5.6–5.9)	0.0401
Current medication, *n*					
ARBs/ACEIs	143 (66.2%)	45 (49.5%)	63 (74.1%)	33 (86.8%)	<0.0001
CCBs	72 (33.3%)	15 (16.5%)	33 (38.8%)	23 (60.5%)	<0.0001
Urine biomarkers					
uTFF1 (*μ*g/gCr)	16.6 (10.5–36.7)	13.1 (7.50–21.5)	17.5 (11.0–39.5)	35.2 (18.4–65.9)	<0.0001
uTFF2 (*μ*g/gCr)	199.7 (146.0–271.4)	204.8 (149.0–289.6)	207.9 (150.0–271.9)	193.3 (127.5–231.1)	0.1903
uTFF3 (*μ*g/gCr)	65.3 (39.3–162.3)	45.6 (28.6–62.8)	70.8 (46.4–142.0)	356.6 (237.4–462.7)	<0.0001
u*α*1-MG (mg/gCr)	3.81 (1.64–9.82)	1.64 (1.03–3.28)	5.29 (2.39–9.42)	27.9 (12.5–49.4)	<0.0001
u*β*2-MG (*μ*g/gCr)	130.9 (68.3–742.2)	97.2 (64.8–155.8)	178.4 (68.3–808.2)	8270 (593.5–34964)	<0.0001
uNAG (U/gCr)	4.75 (3.09–7.42)	3.31 (2.22–5.27)	5.72 (3.97–7.74)	7.09 (4.78–10.4)	<0.0001

ARB, angiotensin receptor blocker; ACEI, angiotensin-converting enzyme inhibitor; CCB, calcium channel blocker; CKD, chronic kidney disease; eGFR, estimated glomerular filtration rate; LDL, low-density lipoprotein; MBP, mean blood pressure; UAE, urinary albumin excretion; u*α*1-MG, urinary *α*1-microglobulin; u*β*2-MG, urinary *β*2-microglobulin; uNAG, urinary N-acetyl-*β*-D-glucosaminidase; and uTFF, urinary trefoil factor. ^*∗*^Chronic glomerulonephritis includes 52 cases (42.3%) of IgA nephropathy, 27 cases (22.0%) of non-IgA mesangial nephritis, 14 cases (11.4%) of lupus nephritis, 13 cases (10.6%) of membranous nephropathy, 8 cases (6.5%) of IgA vasculitis with nephritis, 6 cases (4.9%) of focal segmental glomerulosclerosis, and 3 cases (2.3%) of membranoproliferative glomerulonephritis. ^*∗∗*^Others include 30 cases of unknown etiology without a renal biopsy; 13 cases of anti-neutrophil cytoplasmic antibody-associated vasculitis; 3 cases of polycystic kidney disease, obesity-related glomerulopathy, and solitary kidney; 2 cases of Alport syndrome and vesicoureteral reflux; and 1 case of chronic tubulointerstitial nephritis, cholesterol crystal embolization, light and heavy chain deposition disease, fibrillary glomerulonephritis, crescentic glomerulonephritis with IgA deposit, endocapillary proliferative glomerulonephritis, preeclamptic nephropathy, chronic pyelonephritis, and functional solitary kidney.

**Table 2:** Univariate correlation among uTFF1, uTFF2, uTFF3, and other parameters.

	uTFF1 (*μ*g/gCr) *R* value	uTFF2 (*μ*g/gCr) *R* value	uTFF3 (*μ*g/gCr) *R* value
UAE (mg/gCr)	0.1919	−0.0945	0.2143
eGFR (mL/min/1.73 m^2^)	−0.3302^*∗*^	0.1126	−0.5016^*∗*^
uTFF1 (*μ*g/gCr)	—	0.3910^*∗*^	0.4113^*∗*^
uTFF2 (*μ*g/gCr)	0.3910^*∗*^	—	−0.0109
uTFF3 (*μ*g/gCr)	0.4113^*∗*^	−0.0109	—
u*α*1-MG (mg/gCr)	0.4606^*∗*^	−0.0378	0.7032^*∗*^
u*β*2-MG (*μ*g/gCr)	0.4021^*∗*^	−0.0831	0.5634^*∗*^
uNAG (U/gCr)	0.1920	0.1650	0.2709^*∗*^

eGFR, estimated glomerular filtration rate; UAE, urinary albumin excretion; u*α*1-MG, urinary *α*1-microglobulin; u*β*2-MG, urinary *β*2-microglobulin; uNAG, urinary N-acetyl-*β*-D-glucosaminidase; uTFF, urinary trefoil factor; ^*∗*^*p* <0.0001.

**Table 3:** AUC for predicting the progression of CKD ≥ 3b.

	AUC
Urine biomarkers	
uTFF1 (*μ*g/gCr)	0.750^*∗*^
uTFF2 (*μ*g/gCr)	0.513
uTFF3 (*μ*g/gCr)	0.879^*∗*^
u*α*1-MG (mg/gCr)	0.874^*∗*^
u*β*2-MG (*μ*g/gCr)	0.800^*∗*^
uNAG (U/gCr)	0.674^*∗*^
UAE (mg/gCr)	0.692^*∗*^

AUC, area under the curve; CKD, chronic kidney disease; UAE, urinary albumin excretion, u*α*1-MG, urinary *α*1-microglobulin; u*β*2-MG, urinary *β*2-microglobulin; uNAG, urinary N-acetyl-*β*-D-glucosaminidase; uTFF, urinary trefoil factor; ^*∗*^*p* < 0.005.

**Table 4:** A multiple logistic regression analysis of the predictors of CKD ≥ 3b.

	Odds ratio	95% CI
Urine biomarkers		
uTFF1 > median (*μ*g/gCr)	2.221	0.804–6.364
uTFF2 > median (*μ*g/gCr)	1.188	0.474–3.003
uTFF3 > median (*μ*g/gCr)	3.854^*∗*^	1.316–11.55
u*α*1-MG > median (mg/gCr)	3.958^*∗*^	1.172–14.28
u*β*2-MG > median (*μ*g/gCr)	1.010	0.380–3.013
uNAG > median (U/gCr)	0.862	0.324–2.172
UAE > 300 (mg/gCr)	1.690	0.674–4.276

Adjusted for age, gender, mean blood pressure, uric acid, and renin angiotensin system blockade treatment; CI, confidence interval; CKD, chronic kidney disease; UAE, urinary albumin excretion, u*α*1-MG, urinary *α*1-microglobulin; u*β*2-MG, urinary *β*2-microglobulin; uNAG, urinary N-acetyl-*β*-D-glucosaminidase; uTFF, urinary trefoil factor; ^*∗*^*p* < 0.05.

## Data Availability

The cohort data used in this article contain anonymized but individual data. Therefore, we would prefer not to share this database.

## Abbreviations

*α*1-MG: Alpha 1-microglobulin
ACEIs: Angiotensin-converting enzyme inhibitors
AKI: Acute kidney injury
ARBs: Angiotensin receptor blockers
AUC: Area under the curve
*β*2-MG: Beta 2-microglobulin
CIs: Confidence intervals
CKD: Chronic kidney disease
DBP: Diastolic blood pressure
eGFR: Estimated GFR
ELISA: Enzyme-linked immunosorbent assay
ESRD: End-stage renal disease
GFR: Glomerular filtration rate
MBP: Mean blood pressure
NAG: N-Acetyl-*β*-d-glucosaminidase
ORs: Odds ratios
ROC: Receiver operating characteristic
SBP: Systolic blood pressure
TFF: Trefoil factor family.

## Supplementary Materials

Supplementary Materials Supplementary Table S1: the reproducibility of ELISA system for TFFs. Supplementary Table S2: univariate correlation among uTFFs and age. Supplementary Table S3: the data of uTFFs in healthy subjects. Supplementary Table S4: AUC for predicting the progression of CKD ≥ 3b. Supplementary Table S5: AUC of ratios among parameters for predicting the progression of CKD ≥ 3b. Supplementary Figure S1: the flowchart of inclusion and exclusion criteria in the study. Supplementary Figure S2: the standard curves and the dilution tests of the ELISA system for TFFs. Supplementary Figure S3: box and line plots showing the levels of urine TFF without creatinine correction according to the CKD stages. Supplementary Figure S4: the renal survival categorized by urine TFF alone without creatinine correction (A–C) or by their combination with albuminuria (D–F). Supplementary Figure S5: the levels of urine TFF without creatinine correction for the renal endpoint group and the renal survival group.
