# Supplementary material for: Urine Trefoil Factors as Prognostic Biomarkers in Chronic Kidney Disease
Source: Biomed Res Int. 2018 Apr 3;2018:3024698. doi: 10.1155/2018/3024698 (PMC5903307; doi:10.1155/2018/3024698)
Supplement: Supplementary Materials — Supplementary Table S1: the reproducibility of ELISA system for TFFs. Supplementary Table S2: univariate correlation among uTFFs and age. Supplementary Table S3: the data of uTFFs in healthy subjects. Supplementary Table S4: AUC for predicting the progression of CKD ≥ 3b. Supplementary Table S5: AUC of ratios among parameters for predicting the progression of CKD ≥ 3b. Supplementary Figure S1: the flowchart of inclusion and exclusion criteria in the study. Supplementary Figure S2: the standard curves and the dilution tests of the ELISA system for TFFs. Supplementary Figure S3: box and line plots showing the levels of urine TFF without creatinine correction according to the CKD stages. Supplementary Figure S4: the renal survival categorized by urine TFF alone without creatinine correction (A–C) or by their combination with albuminuria (D–F). Supplementary Figure S5: the levels of urine TFF without creatinine correction for the renal endpoint group and the renal survival group. [file 3024698.f1.pdf]

Urine Trefoil Factors as Prognostic Biomarkers in Chronic Kidney Disease

Toshio Yamanari, Hitoshi Sugiyama, Keiko Tanaka, Hiroshi Morinaga, Masashi Kitagawa, Akifumi Onishi, Ayu Ogawa-Akiyama, Yuzuki Kano, Koki Mise, Yasukazu Ohmoto, Kenichi Shikata, and Jun Wada

SUPPLEMENTARY INFORMATION

Table S1. The reproducibility of ELISA system for TFFs

TFF1

Intra-assay	Sample 1	Sample 2	Sample 3	Sample 4
Number of replicates	5	5	5	5
Mean (ng/mL)	0.024	0.086	0.094	0.347
SD	0.001	0.004	0.005	0.016
CV (%)	4.16	4.79	4.96	4.66

Inter-assay	Sample 1	Sample 2	Sample 3	Sample 4
Number of determinations	4	4	4	4
Mean (ng/mL)	0.024	0.091	0.100	0.354
SD	0.001	0.003	0.004	0.014
CV (%)	3.42	2.92	3.95	3.83

TFF2

Intra-assay	Sample 1	Sample 2	Sample 3	Sample 4
Number of replicates	5	5	5	5
Mean (ng/mL)	0.421	1.180	1.249	6.806
SD	0.021	0.046	0.041	0.152
CV (%)	4.94	3.90	3.29	2.23

Inter-assay	Sample 1	Sample 2	Sample 3	Sample 4
Number of determinations	4	4	4	4
Mean (ng/mL)	0.419	1.200	1.235	6.723
SD	0.021	0.049	0.054	0.280
CV (%)	4.94	4.10	4.36	4.16

TFF3

Intra-assay	Sample 1	Sample 2	Sample 3	Sample 4
Number of replicates	5	5	5	5
Mean (ng/mL)	0.134	0.484	0.595	1.722
SD	0.006	0.020	0.028	0.078
CV (%)	4.72	4.17	4.65	4.53

Inter-assay	Sample 1	Sample 2	Sample 3	Sample 4
Number of determinations	4	4	4	4
Mean (ng/mL)	0.137	0.500	0.595	1.722
SD	0.004	0.018	0.026	0.075
CV (%)	2.65	3.65	4.34	4.34

TFF, trefoil factor family; SD, standard deviation; CV, coefficient of variation

Table S2. Univariate correlation among uTFFs and age

	R value	p value
uTFF1 (μg/gCr)	0.3433	< 0.0001
uTFF2 (μg/gCr)	0.2041	0.0026
uTFF3 (μg/gCr)	0.2549	0.0002

uTFF, urinary trefoil factor

Table S3. The data of uTFFs in healthy subjects

N = 6 (Male = 3)		Lower limitation	Upper limitation
uTFF1 (μg/gCr)	8.40 (4.28-40.1)	2.81	87.5
uTFF2 (μg/gCr)	343 (284-646)	263	1104
uTFF3 (μg/gCr)	30.9 (17.6-36.6)	15.0	39.1

uTFF, urinary trefoil factor

Table S4. AUC for predicting the progression of CKD ≥ 3b

	AUC
Clinical parameters	
Age (years)	0.725*
MBP (mmHg)	0.512
Hemoglobin (g/dL)	0.752*
Uric acid (mg/dL)	0.751*
Hemoglobin A_{1C} (%)	0.563

AUC, area under the curve; CKD, chronic kidney disease; MBP, mean blood pressure

*P <0.0001

Table S5. AUC of ratios among parameters for predicting the progression of CKD ≥ 3b

	AUC
Urine biomarkers	
Ratio of uTFF3/Cr to uTFF1/Cr	0.686*
Ratio of uTFF3/Cr to uTFF2/Cr	0.859*
Ratio of uTFF3/Cr to UAE	0.480
Ratio of uTFF3/Cr to uα1-MG/Cr	0.658*
Ratio of uTFF3/Cr to uβ2-MG/Cr	0.314
Ratio of uTFF3/Cr to uNAG/Cr	0.782*

AUC, area under the curve; CKD, chronic kidney disease; UAE, urinary albumin excretion; uα1-MG, urinary α1-microglobulin; uβ2-MG, urinary β2-microglobulin; uNAG, urinary N-acetyl-β-D-glucosaminidase; uTFF, urinary trefoil factor

*P < 0.05

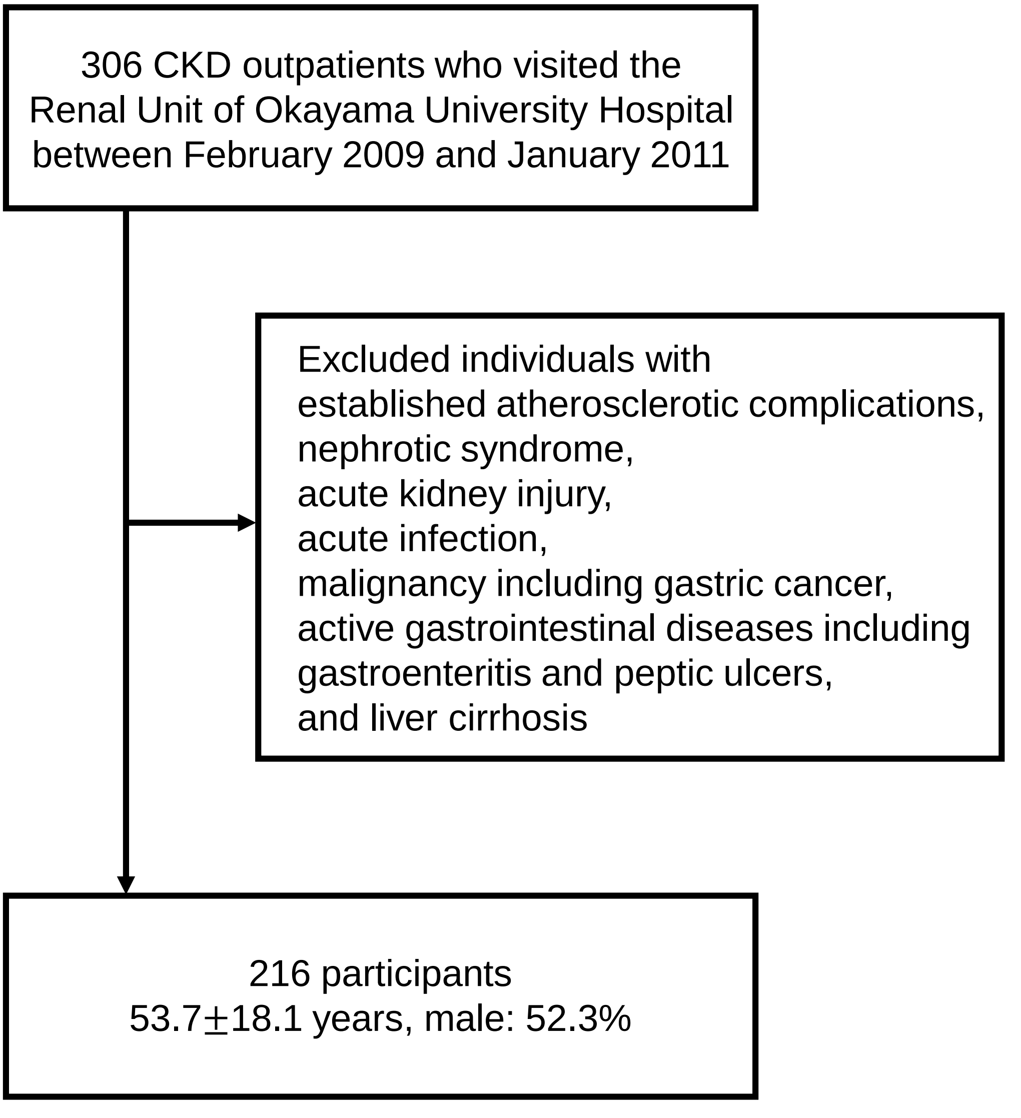

Figure S1. The flow chart of inclusion and exclusion criteria in the study.

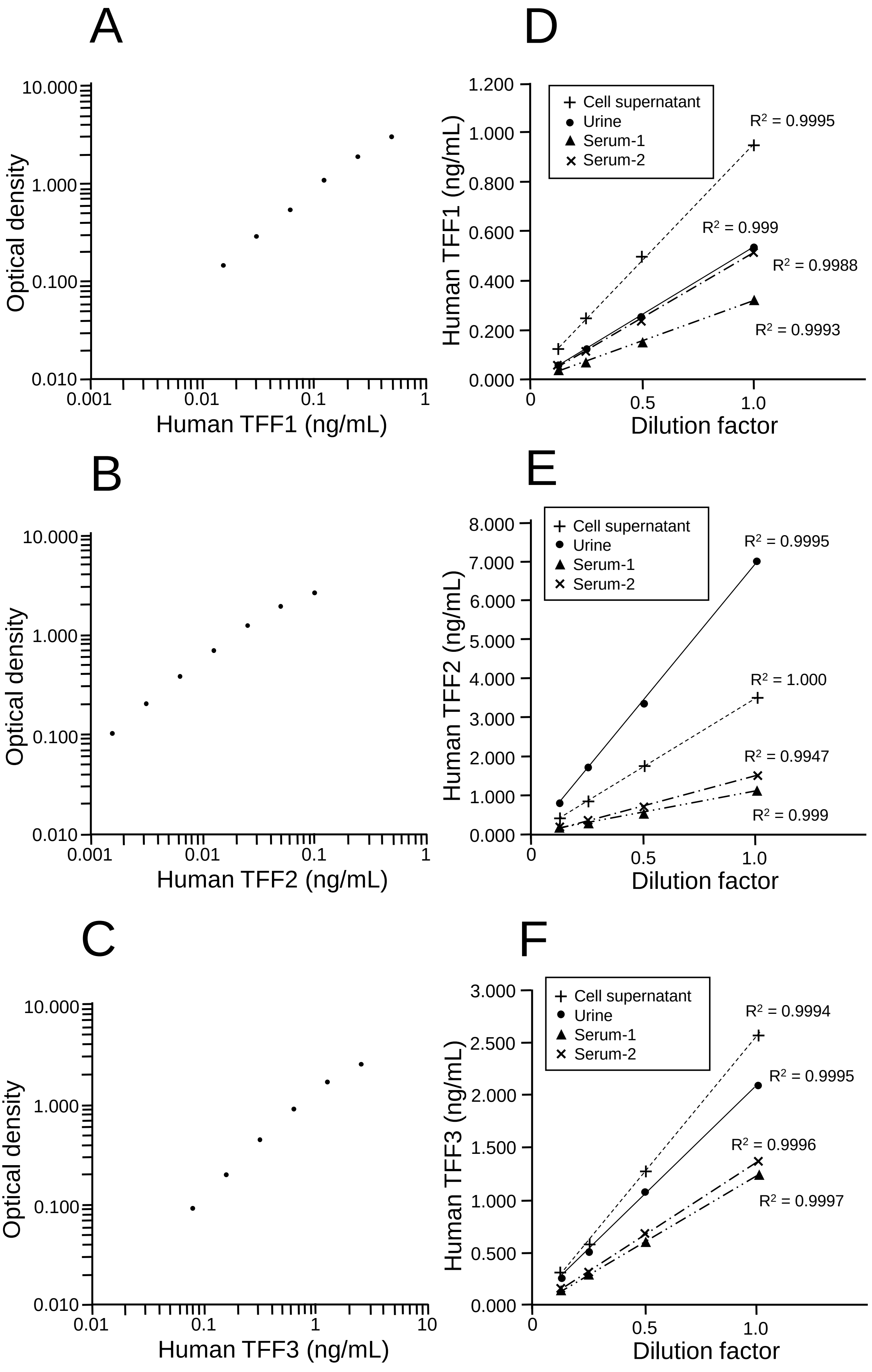

Figure S2. The standard curves and the dilution tests of the ELISA system for TFFs. The standard curves of TFF1 (A), TFF2 (B) and TFF3 (C) were plotted by logarithmic scales. The dilution tests of TFF1 (D), TFF2 (E) and TFF3 (F) were analyzed with samples of cell supernatant, serum and urine.

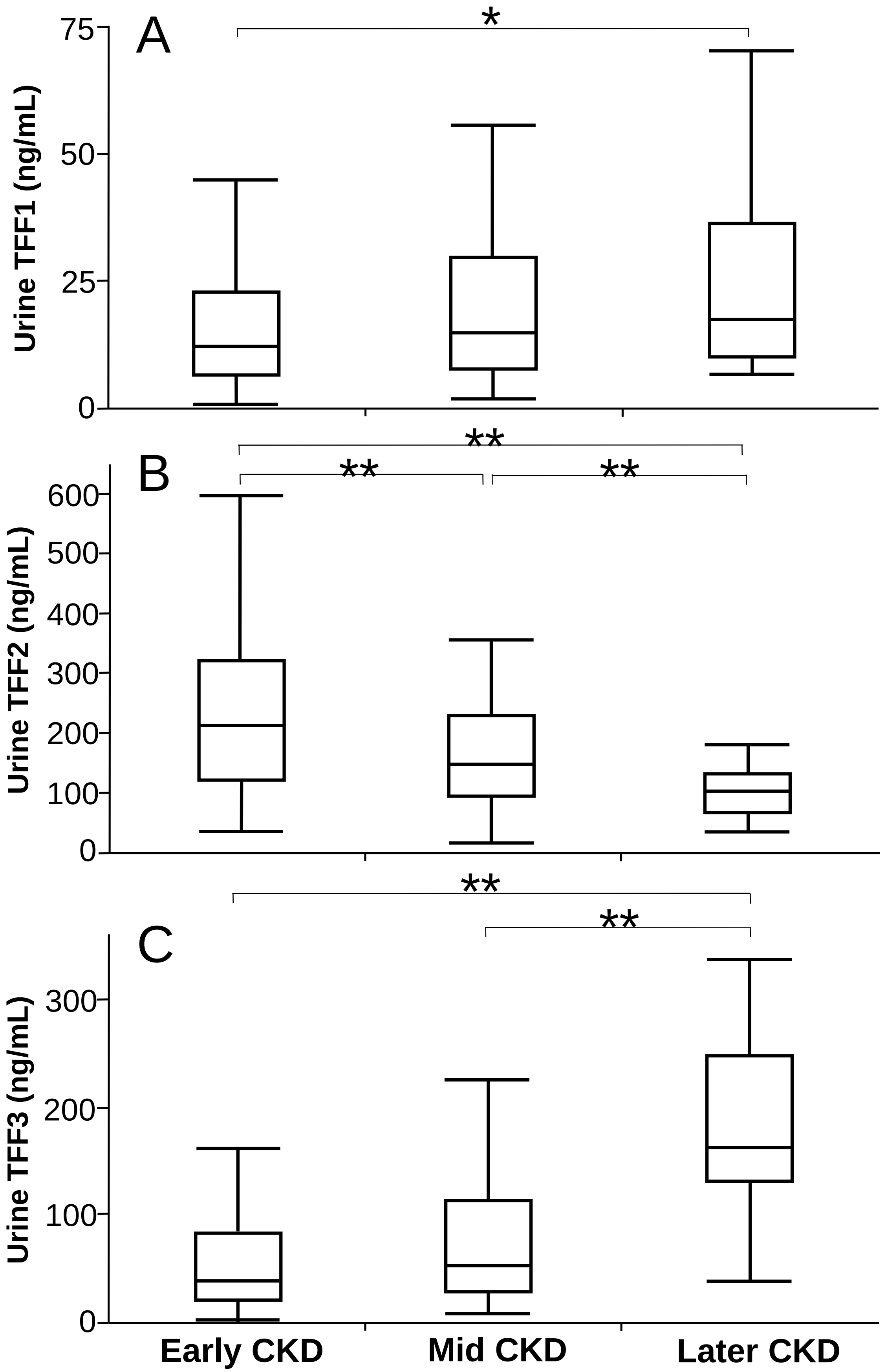

Figure S3. Box and line plots showing the levels of urine TFF without creatinine correction according to the CKD stages. The levels of both urine TFF1 (ng/mL) (A) and TFF3 (ng/mL) (C) increased with CKD stage, while those of urine TFF2 (ng/mL) decreased with CKD stage (B). * and ** indicate $p < 0.005$ and $p < 0.0001$, respectively. The boxes denote the medians and 25th and 75th percentiles. The lines mark the 5th and 95th percentiles.

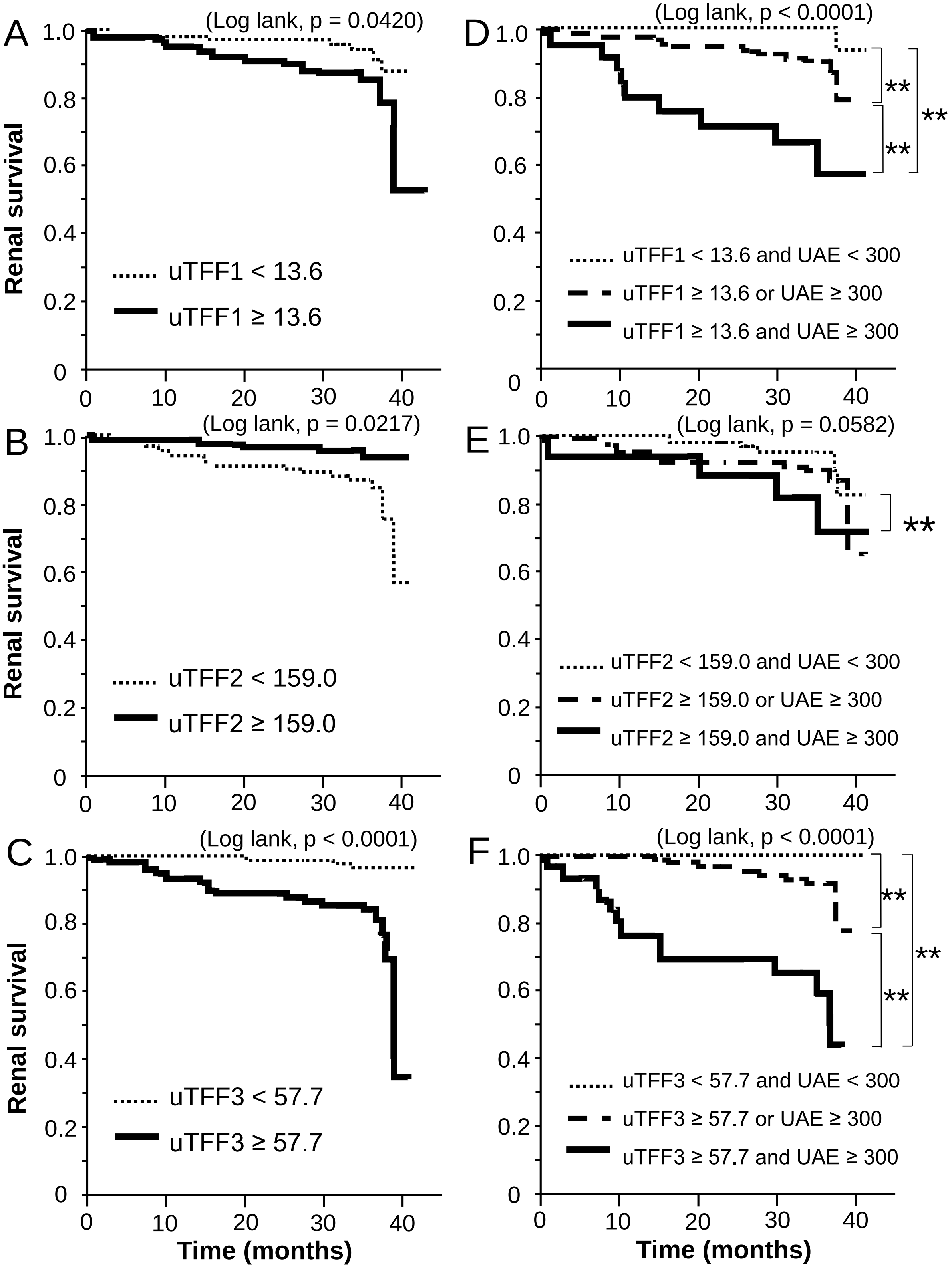

Figure S4. The renal survival categorized by urine TFF alone without creatinine correction (A-C) or by their combination with albuminuria (D-F). The median value of urine TFF1 (ng/mL) (A), urine TFF2 (ng/mL) (B) and TFF3 (ng/mL) (C) predicted the three-year renal endpoint-free survival. The combination of urine TFF1 (D) or TFF3 (F) with albuminuria clearly separated the three-year renal endpoint-free survival of CKD patients, while that of urine TFF2 with albuminuria had a less obvious effect (E). (D) uTFF1 < 13.6 and UAE < 300, n = 80 (37.0%), uTFF1 ≥ 13.6 or UAE ≥ 300, n = 110 (51.0%), uTFF1 ≥ 13.6 and UAE ≥ 300, n = 26 (12.0%). (E) uTFF2 < 159.0 and UAE < 300, n = 71 (32.9%), uTFF2 ≥ 159.0 or UAE ≥ 300, n = 126 (58.3%), uTFF2 ≥ 159.0 and UAE ≥ 300, n = 19 (8.8%). (F) uTFF3 < 57.7 and UAE < 300, n = 86 (39.8%), uTFF3 ≥ 57.7 or UAE ≥ 300, n = 99 (45.8%), uTFF3 ≥ 57.7 and UAE ≥ 300, n = 31 (14.4%). * and ** indicate p < 0.01 and p < 0.0001, respectively. UAE, urinary albumin excretion (mg/gCr).

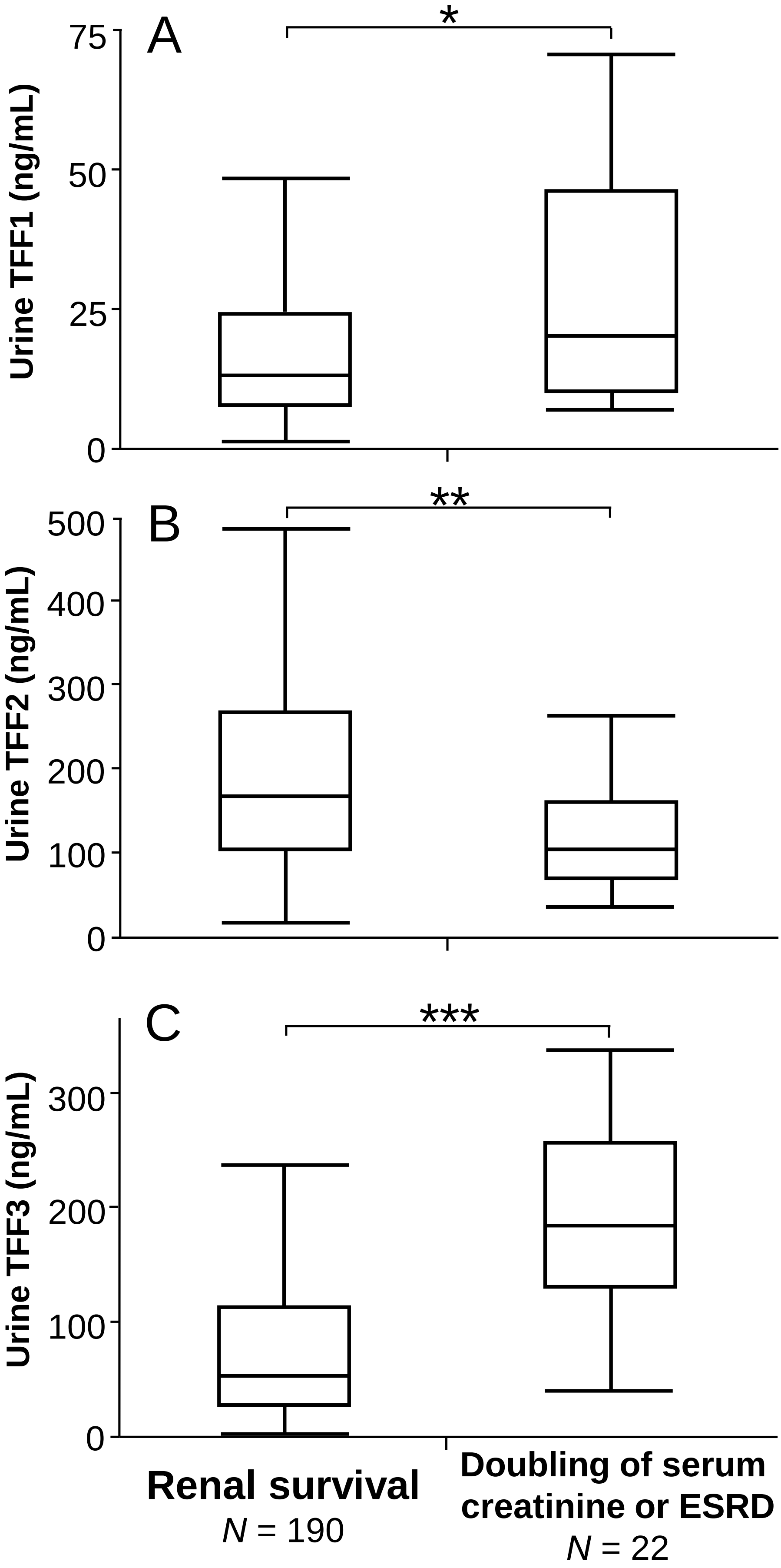

Figure S5. The levels of urine TFF without creatinine correction for the renal endpoint group and the renal survival group. The renal endpoint group (right box and line plot) had higher levels of urine TFF1 (A) and TFF3 (C) but lower levels of urine TFF2 than the renal survival group (B). *, **, and *** indicate $p < 0.05$, $p < 0.001$ and $p < 0.0001$, respectively. The boxes denote the medians and 25th and 75th percentiles. The lines mark the 5th and 95th percentiles.
